# Excess Mortality on Italian Small Islands during the SARS-CoV-2 Pandemic: An Ecological Study

**DOI:** 10.3390/idr14030043

**Published:** 2022-05-26

**Authors:** Matteo Riccò, Pietro Ferraro, Simona Peruzzi, Alessandro Zaniboni, Elia Satta, Silvia Ranzieri

**Affiliations:** 1 Servizio di Prevenzione e Sicurezza Negli Ambienti di Lavoro (SPSAL), AUSL-IRCCS di Reggio Emilia, Via Amendola n.2, I-42122 Reggio Emilia, Italy; 2 Occupational Medicine Unit, Direzione Sanità, Italian Railways’ Infrastructure Division, RFI SpA, I-00161 Rome, Italy; dott.pietro.ferraro@gmail.com; 3 Laboratorio Analisi Chimico Cliniche e Microbiologiche, Ospedale Civile di Guastalla, AUSL-IRCCS di Reggio Emilia, Via Donatori di Sangue n.1, I-42016 Guastalla, Italy; simona.peruzzi@ausl.re.it; 4 Department of Medicine and Surgery, University of Parma, I-43126 Parma, Italy; alessandro.zaniboni@unipr.it (A.Z.); elia.satta@unipr.it (E.S.); silvia.ranzieri@unipr.it (S.R.)

**Keywords:** case fatality rate, Coronavirus, islands, Malta, mortality, syndemic, mortality rates, excess mortality rates

## Abstract

Small islands have been considered at an advantage when dealing with infectious diseases, including COVID-19, but the evidence is still lacking. Crude mortality rates (CMRs) and excess mortality rates (EMRs) were calculated for 35 municipalities on the Italian small islands for 2020 and 2021, and the corresponding estimates were compared to those of the parent provinces and the national estimates. Notification rates for COVID-19 were retrieved, but detailed data at the municipality level were not available. A relatively low CMR (1.069 per 100 per year, 95% confidence interval [95% CI] 0.983–1.164) was identified in 2020, compared to 1.180, 95% CI 1.098–1.269 for 2021. EMRs of small islands ranged between −25.6% and +15.6% in 2020, and between −13.0% and +20.9% in 2021, with an average gain of +0.3% (95% CI −5.3 to +5.8) for the entirety of the assessed timeframe, and no substantial differences between 2020 and 2021 (pooled estimates of −4.1%, 95% CI −12.3 to 4.1 vs. 4.6%, 95% CI −3.1 to 12.4; *p* = 0.143). When dealing with COVID-19 notification rates, during the first wave, parent provinces of Italian small islands exhibited substantially lower estimates than those at the national level. Even though subsequent stages of the pandemic (i.e., second, third, and fourth waves) saw a drastic increase in the number of confirmed cases and CMR, estimates from small islands remained generally lower than those from parent provinces and the national level. In regression analysis, notification rates and mortality in the parent provinces were the main effectors of EMRs in the small islands (β = 0.469 and β = 22.768, *p* < 0.001 and *p* = 0.007, respectively). Contrarily, the management of incident cases in hospital infrastructures and ICUs was characterized as a negative predictor for EMR (β = −11.208, *p* = 0.008, and −59.700, *p* = 0.003, respectively). In summary, the study suggests a potential role of small geographical and population size in strengthening the effect of restrictive measures toward countering the spread and mortality rate of COVID-19.

## 1. Introduction

Islands may be defined as territories having a minimum surface of 1 km^2^, a minimum distance between the island and the mainland of 1 km, a resident population of more than 50 inhabitants, and no fixed link (for example, a bridge, a tunnel, or a dyke) between the land mass and the mainland [1,2]. It is a quite vague definition, encompassing both large and very small land masses, with similarly diverse population sizes. In 1990, Brookfield suggested classifying Pacific islands by their surface area and population scales, and such classifications have been employed even in other settings, such as the Mediterranean Sea [3,4]. According to this classification, islands with a total surface area <1000 km^2^ and less than 100,000 inhabitants may be recognized as minor or small islands.

Since the inception of the SARS-CoV-2 pandemic, it was hypothesized that the small geographical and population sizes of small islands, coupled with the appropriate implementation of non-pharmaceutical interventions (NPIs) recommended by international authorities [5], could be an advantage in the effort to contain viral spread and maintain a low mortality rate [6]. On the other hand, small islands may be unprepared to respond efficiently to a pandemic because of the limited availability of health resources [7]. Moreover, small islands are often involved in tourism and migratory routes: particularly in the earlier stages of the pandemic, the influx of individuals from areas where the virus was active may have provided a fertile ground for its uncontrolled spread [7,8,9,10].

However, scarce evidence has been collected, and mostly from small island states such as Malta and Cyprus, whose size and resources still appear as not comparable to those of small islands [6,8,9,10].

From this point of view, Italy may provide some interesting insights. In fact, the Italian peninsula includes two major islands (Sicily and Sardinia), and 30 small islands for a total of 35 administrative units. The pooled surface area of Italian small islands totals 874.41 km^2^ (0.29% of total Italian surface area) and serves a total population of around 200,000 individuals (i.e., 0.3% to 0.4% of the entire Italian population) [11]. To date, no studies have specifically examined the impact of the SARS-CoV-2 pandemic on these areas: in order to fill this information gap, this study, therefore, compared mortality data from all cases over 24 months (January 2020 to December 2021) in the 35 administrative units from Italian small islands. More precisely, we assessed estimates for excess mortality rates (EMRs) and their main effectors, as they could provide information about the burden of mortality related to the pandemic, including deaths indirectly associated with it, overcoming the potential ambiguities (i.e., deaths “with” COVID vs. “due to” COVID) and the nuances of the diverse demographics of the small islands [12,13].

## 2. Materials and Methods

### 2.1. Background

Italy has a total surface area of 301,230 km^2^, with a total population of around 60,000,000 inhabitants. The first-level administrative entities are represented by the Regions (20); each region is divided into a number of provinces (107, including 80 ordinary provinces, 2 autonomous provinces, 4 regional decentralization entities, 6 free municipal consortia, 14 metropolitan cities, as well as the Aosta Valley Region, where region and province coincide) that in turn include several municipalities (in Italian, “comune”; totaling 7904 as of 18 March 2022), that vary considerably in size and population, both in terms of total population and population density [14].

**Figure A1:**
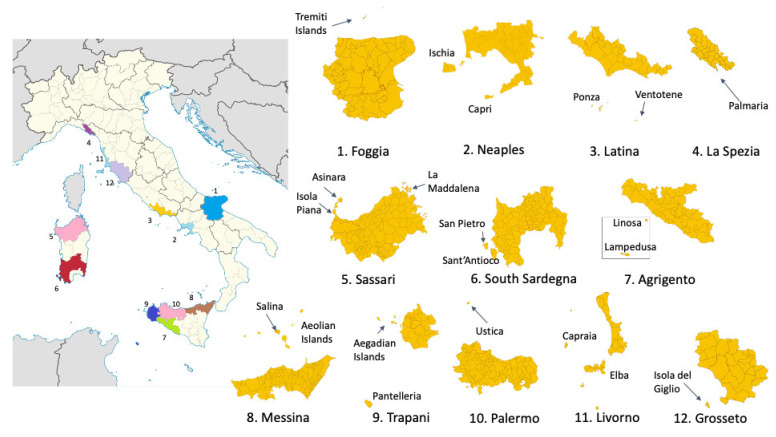
Italian small islands and their respective parent provinces.

The main land mass is represented by the Italian peninsula, with two major Mediterranean islands (Sicily and Sardinia, with total surface areas of 25,711 km^2^ and 24,090 km^2^, respectively, and total populations of 4,875,290 and 1,611,621 inhabitants, respectively, in 2020), and a total of 30 Italian small islands (see Table A1; Figure A1). Italian small islands are organized into 35 municipalities from 12 provinces and seven regions (Campania, Latium Liguria, Apulia, Sardinia, Sicily, Tuscany), with large heterogeneities. For example, the largest among the Italian small islands (i.e., Isola d’Elba; 224 km^2^) encompasses a total of eight municipalities, while the Aeolian Islands of Lipari, Vulcano, Salina, Stromboli, Filicudi, Alicudi, and Panarea are organized into a single municipality (Lipari), and similarly, the scarcely populated Tremiti Islands (San Domino, San Nicola, Capraia, Cretaccio, Pianosa) constitute a single municipality of the province of Foggia.

### 2.2. Population Data

Demographic data were obtained from the official website of the Italian Institute of Statistics (ISTAT; https://demo.istat.it, accessed on 20 March 2022). Open-source data included population estimates at various administrative levels (i.e., national, regional, provincial and/or municipality). More precisely, we retrieved monthly estimates from the timeframe 2015–2021 about: (a) resident population at the provincial and municipality levels; (b) monthly estimates for deaths among residents at the provincial and municipality levels; and (c) income for the general population and renters.

### 2.3. Data about COVID-19

The official website of the Italian National Institute for Nuclear Physics (INFN) provides open-source provincial-level estimates for SARS-CoV-2 infections, including: (a) notified cases; (b) COVID-19 related deaths; (c) COVID-19 related hospital admissions; (d) COVID-19 related admissions to intensive care units (ICUs). Data about incident cases 2020–2021 were retrieved at the lowest available administrative unit level (i.e., province).

### 2.4. Statistical Analysis

We performed a descriptive analysis of the data by calculating the corresponding annual crude mortality rates of the municipalities from the small islands for the time periods 2015–2019, 2020 and 2021. Municipality-level estimates (i.e., estimates from the individual communities included in the small islands) were then pooled at the provincial level (i.e., pooled estimates of all small-island communities administratively included in the very same province) in order to compare them with general data from the parent province EMR. The difference between the reported number of deaths (RD) in a given month in 2020 and 2021 and the estimate of the expected deaths (ED) for that period was then calculated as follows:

EMR = (RD^i^ − ED^i,a, 2015−2019^)/ED^i,a, 2015−2019^ (1)

RD^i,a^ = reported deaths in a given month i, at a specific administrative level a.

ED^i,a, 2015−2019^ = average deaths in a given month *i* for the time period 2015–2019, at a specific administrative level a.

Average monthly estimates for COVID-19 notification rates, deaths, hospital admissions, and admissions in intensive care units (ICUs) were then calculated at provincial level in order to compare corresponding estimates with mortality and EM from the small islands municipalities pooled at the provincial level. Comparisons of crude rates were performed by means of Mann–Whitney or Kruskal–Wallis test for multiple independent samples. On the other hand, association between continuous variables was assessed through Spearman’s rank correlation coefficient. Eventually, the relationship between the excess mortality rates and demographic factors was investigated through a regression analysis model that included as the outcome variable the monthly mortality rates (assessed at provincial level). The explanatory variables were represented by COVID-19 related factors such as: notification rates, mortality, hospital admissions, and ICU admissions. All variables were entered in the model as a single step. The analyses were then adjusted by main demographic factors (i.e., male to female ratio; share of population aged 60 years or more; annual income).

Eventually, a sensitivity analysis was performed in order to assess how vulnerable the results of regression analysis were to many or all unobserved confounders. In the analyses, we assumed COVID-19 mortality rates as the “treatment” (i.e., the main effector), as we assumed that a large share of incident cases may have been not reported, particularly during the earlier stages of the pandemic [15]. Similarly, according to the available data on COVID-19 mortality stressing high mortality rates in older individuals [15,16], the share of population aged 60 years or more of the small islands was included in the model as the main benchmark confounding factor.

All calculation were performed in R 4.0.3 [17], by means of packages epiR (v. 2.0.19), EpiReport (v 1.0.1), fmsb (0.7.0), sensmakr (0.1.4).

*Ethical approval*. No ethical approval was needed for this study, as no individual data were identifiable, and only aggregated data were analyzed and presented.

## 3. Results

### 3.1. Demographics

As shown in Table 1, the total population of Italian small islands decreased from 218,038 inhabitants in 2015 to 213,093 in 2021 (−2.3%), similarly to the Italian general population (Spearman’s rho = 0.964; *p* = 0.003).

Compared to the Italian general population, the small islands were also characterized by a lower share of individuals aged less than 20 years (pooled estimate of 17.0% vs. 18.2%), a similar share of residents aged 20 to 39 years (22.3% vs. 22.7%), an increased proportion of age groups 40 to 59 years (31.2% vs. 30.5%) and 60 to 79 years (22.8% vs. 22.0%), and a reduced share of residents aged 80 years of older (6.2% vs. 7.0%; chi-square = 220,189, *p* < 0.001). Moreover, the proportion of individuals older than 40 years increased more steadily on the small islands (from 58.2% in 2015 to 62.1% in 2021) than in the general population (chi-square test for trend 158.6, *p* < 0.001). Interestingly, Italian small islands were characterized by an increased share of individuals of male gender than that reported at the national level (pooled proportion of 49.8% vs. 47.5%, chi-square = 3187, *p* < 0.001).

Between 2015 and 2021, a total of 4,627,795 deaths were reported at the national level, with 15,569 of them occurring on the small islands (0.3%), with a substantial increase from 2015 to 2021 (+9.5% at national level vs. +9.2% for small islands; *p* < 0.001).

Yearly mortality rates of municipalities from Italian small islands are reported in Table 2. As shown, pooled all-cause mortality was estimated at 1.105 per 100 per year (95% CI 1.034 to 1.181) for the timeframe 2015–2019, compared to 1.069 (95% CI 0.983 to 1.164) in 2020, and 1.180 (95% CI 1.098 to 1.269) in 2021. However, pooled estimates were affected by substantial heterogeneity (I^2^ = 92.2%, 95% CI 90.1% to 93.8% for 2015–2019; I^2^ = 70.3%, 95% CI 58.1% to 78.9% for 2020; and I^2^ = 63.6%, 95% CI 47.8% to 74.6% for 2021).

**Figure 1:**
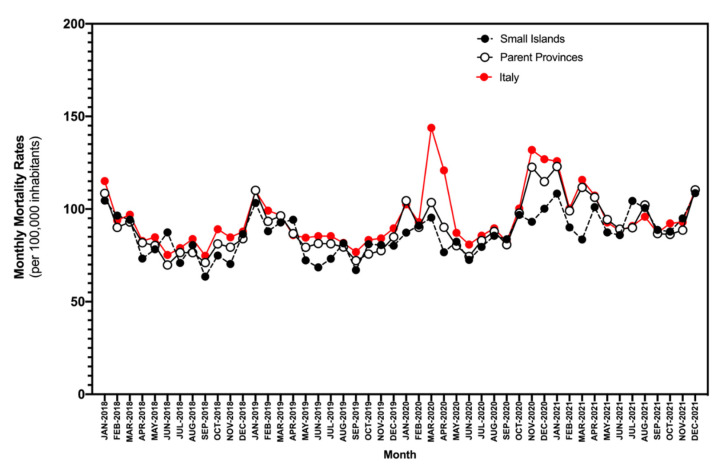
Monthly mortality rates (per 100,000) for Italian small islands, parent provinces, and national level (January 2018–December 2021).

Additionally, monthly mortality rates for 2015–2021 from the 35 municipalities were quite heterogenous (Figure 1), ranging from 63.5 to 104.6 per 100,000 in the pre-pandemic time period (2015–2019), between 76.7 and 100.2 per 100,000 during 2020, and between 86.0 and 108.4 per 100,000 during 2021. When compared to the estimates for 2015–2019, no substantial differences were identified with rates for 2020 (*p* = 0.130) and 2021 (*p* = 0.753). Moreover, monthly mortality rates of the small islands across all of the assessed timeframes were substantially lower than those of the parent provinces (86.4 per 100,000 persons, 95% CI 83.1 to 89.8 vs. 90.2 per 100,000 persons, 95% CI 86.3 to 94.1, *p =* 0.010), and those of the national estimates as well (94.4 per 100,000, 95% 90.4 to 99.4, *p* < 0.001).

**Figure 2:**
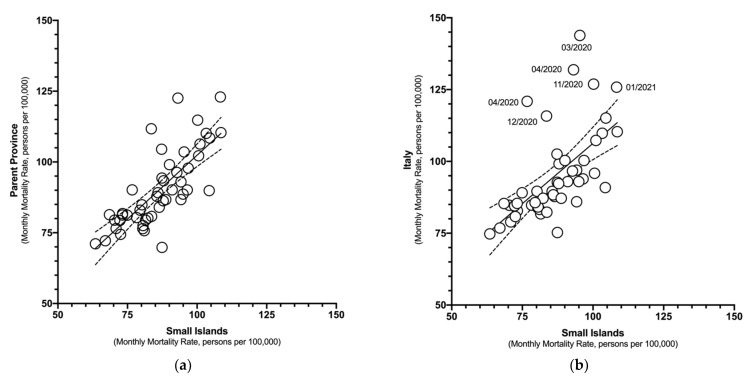
Correlation between monthly mortality estimates for Italian small islands and (**a**) parent provinces (January 2015–December 2021) (Spearman’s rank test rho = 0.783, *p* < 0.001); (**b**) Italy as a whole (Spearman’s rank test rho = 0.728, *p* < 0.001).

Mortality rates on the small islands were positively correlated with corresponding estimates both for parent provinces (Figure 2a; rho = 0.783, *p* < 0.001) and the national level (Figure 2b; rho = 0.728, *p* < 0.001). When focusing on the estimates from 2020 (rho = −0.344) and 2015–2019 (rho = −0.339), a negative correlation with population density (i.e., the higher the population density, the lower the mortality rates in the assessed areas) was suggested, but the results were on the edge of statistical significance (*p* = 0.043 and *p* = 0.046, respectively), and should be cautiously assessed. In fact, no significant correlation was found for 2021 (rho = −0.272, *p* = 0.114).

When crude mortality rates from the small islands in 2020 and 2021 were compared to the background estimates for 2015–2019, no substantially increased risk was identified for 2020 (risk ratio [RR] 0.992, 95% CI 0.933 to 1.056), while it was substantially increased in 2021 (RR 1.096, 95% CI 1.049 to 1.145) (Table 3). In both cases, the heterogeneity of the estimates was substantial (i.e., I^2^ = 70.3%, 95% CI 58.1% to 78.9% for 2020; I^2^ = 63.6%, 95% CI 47.8% to 74.6% for 2021).

Contrarily, when mortality estimates were compared to those from the parent province (Table 4), no substantial difference were identified for 2020 (RR 0.939, 95% CI 0.868 to 1.756), or 2021 (RR 0.992, 95% CI 0.943 to 1.044). Heterogeneity was moderate for 2021 (i.e., I^2^ 34.1%, 95% CI 0.0% to 66.8%), and substantial for 2020 (i.e., I^2^ 60.5%, 95% CI 25.7% to 79.0%).

### 3.2. Characteristics of COVID-19 Pandemic

**Figure 3:**
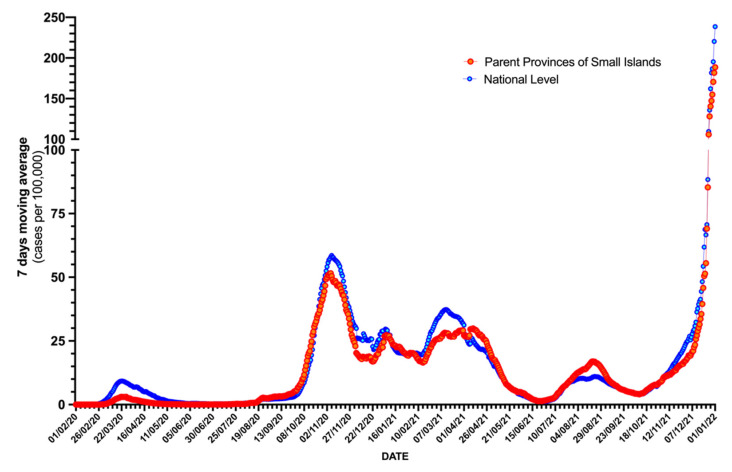
Notification rates (7-day moving average) for provinces including the communities of Italian small islands, and national level estimates (1 February 2020; 31 December 2021).

Detailed estimates of COVID-19 notification rates for the small islands are, to date, not available. The most reliable proxy (i.e., notification rates in parent provinces) is reported in Figure 3 with corresponding estimates for the national level. Even though the epidemic curves were well-correlated from the earlier stages of the pandemic (time period 1 February 2020 to 30 April 2020; rho = 0.982, *p* < 0.001), parent provinces of the small islands exhibited substantially lower notification rates (7-day moving averages for 1.0 per 100,000 inhabitants, 95% CI 0.8 to 1.2 vs. 3.9, 95% CI 3.2 to 4.6; *p* < 0.001). During the subsequent stages of the pandemic, parent provinces of small islands were more extensively involved, still exhibiting lower notification rates (7-day moving averages for 1 September 2020 to 30 June 2021: 20.2 per 100,000 inhabitants, 95% CI 18.7 to 21.6 vs. 21.8 per 100,000 inhabitants, 95% CI 20.1 to 23.5, *p* < 0.001), with the notable exception of the month of August 2021.

**Figure 4:**
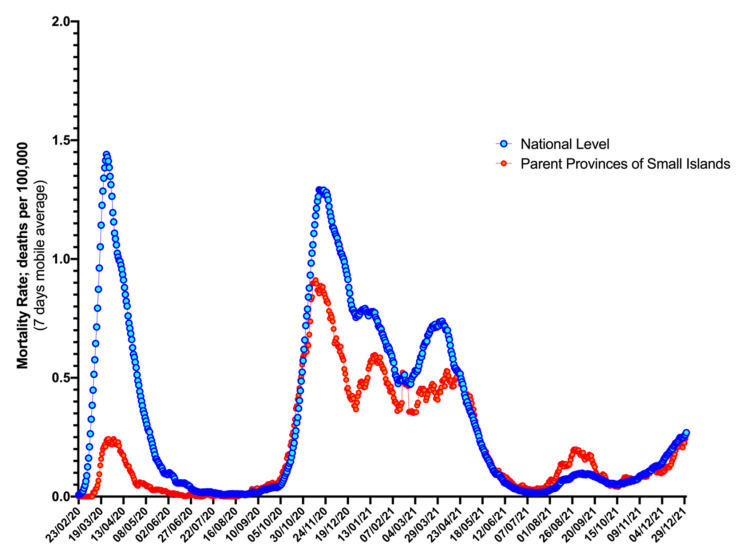
Mortality rates (7-day moving average) in parent provinces of Italian small islands, and at national level (25 February 2020; 31 December 2021).

A similar pattern was identified even when COVID-19 associated mortality rates were taken into account (Figure 4). As shown, parent provinces of small islands had substantially lower rates than those reported at the national level for the time period from 1 February 2020 to 30 April 2020, while national level estimates calculated for the entire Italian population peaked at 1.4 per 100,000 (7-day moving average: 0.7 per 100,000 inhabitants, 95% CI 0.6 to 0.8); in parent provinces of small islands, the daily peak did not exceed 0.2 per 100,000 inhabitants (7-day moving average: 0.1 per 100,000, 95% CI 0.1 to 0.2; *p* < 0.001). Similarly to notification rates, in the subsequent stages of the pandemic, the trend remained quite similar (Spearman’s rho = 0.835, *p* < 0.001), but the differences remained significantly different (3.3 per million inhabitants, 95% CI 2.9 to 3.6 vs. 2.6 per million inhabitants, 95% CI 2.4 to 2.8, *p* < 0.001).

A positive correlation between the number of COVID-19 positive cases and mortality rates in the parent provinces was expected as a result of the high infectivity rate and morbidity. However, no actual correlation was identified (rho = 0.094; *p* = 0.141). Contrarily, general mortality on the small islands was positively correlated with COVID-19 associated mortality in the parent provinces (rho = 0.118; *p* = 0.037). Hospital admission rates and ICU admission rates were also compared, but while hospital admissions for COVID-19 (rho = 0.179; *p* = 0.005) were positively correlated with the mortality rates, the latter were not clearly correlated with ICU admission rates (rho = 0.120; *p* = 0.062).

### 3.3. Excess Mortality

**Figure 5:**
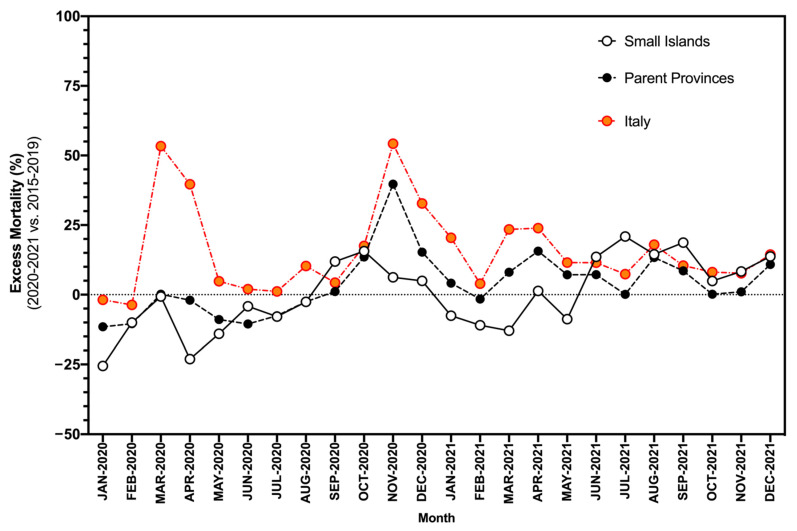
Excess mortality on Italian small islands compared to parent provinces and national estimates for the time period January 2020–December 2021. In the calculations, average monthly mortality rates for 2015–2019 were retained as references.

Municipality level estimates for EMRs on the small islands ranged between −25.6% and +15.6% in 2020, and between −13.0% and +20.9% in 2021 (Figure 5), with an average of +0.3% (95% CI −5.3 to +5.8) for the entirely of the assessed timeframe, and no substantial differences between 2020 and 2021 (−4.1%, 95% CI −12.3 to 4.1 vs. 4.6%, 95% CI −3.1 to 12.4; *p* = 0.143). In this regard, visual inspection of Figure 4 reveals a substantial outlier represented by the months of March and April 2020 (i.e., the “first wave”), when national estimates substantially exceeded those for the small islands and their parent provinces. A similar outlier was identified in the second half of 2020 (i.e., the “second wave”) that still had a limited impact on the small islands.

**Figure 6:**
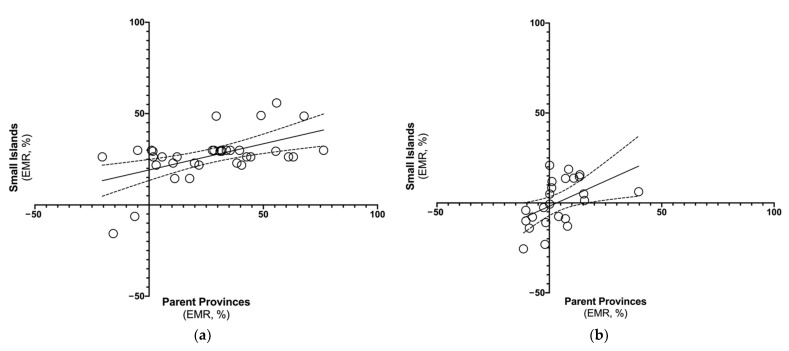
Correlation of excess mortality rates (EMRs) on Italian small islands (2020–2021) with estimates for parent provinces: (**a**) as individual estimates (rho = 0.416, *p* = 0.013), (**b**) pooled estimates by calendar month (rho = 0.592, *p* = 0.002).

Both individual (Figure 6a) and pooled monthly EMR estimates for the small islands (Figure 6b) were positively correlated with the estimates for the parent provinces (respectively: rho = 0.416, *p* = 0.013, and rho = 0.592, *p* = 0.002). However, pooled estimates were unrelated with the national level (rho = 0.351, *p* = 0.351). EMR was also unrelated with population density (rho = 0.231, *p* = 0.181), and with demographic factors such as the share of individuals aged 60 years or more (rho = −0.007, *p* = 0.966), or 80 years or more (rho = 0.055, *p* = 0.754), or average per capita income (rho = 0.277, *p* = 0.108).

### 3.4. Regression Analysis

In regression analysis, monthly EMRs on Italian small islands were included in a multivariable model where COVID-19 notification rates, COVID-19 related mortality, and admissions (both as a whole and in ICUs) were assessed as effector variables. The model was adjusted for main demographic factors (i.e., age groups; gender; annual income). As summarized in Table 5, mortality rates were higher when notification rates (β 0.469, B 0.882, 95% CI 0.551 to 1.212), and mostly COVID-19 related mortality (β 22.768, B 8.385, 95% CI 6.285 to 39.250) were increased.

Conversely, admission estimates for COVID-19—both in general (β −11.208, B 3.278, 95% CI −17.651 to −4.765), and to ICUs—were characterized as negative effectors (β −59.700, B 19.710, 95% CI −98.444 to −20.956) for EMRs on the small islands. In other words, EMRs were higher when notification rates and mortality associated with SARS-CoV-2 infections in the parent provinces were higher, while they were decreased by higher admissions in hospital infrastructures, both in general and in ICUs.

In sensitivity analysis, the robustness value for bringing the point estimate of EMR exactly to zero (i.e., RV q = 1) was 13.2%. In other words, unobserved confounders that explained 13.2% of the residual variance from the treatment variable (i.e., COVID-19 mortality) and outcome variable were sufficient to explain all of the observed effects. On the other hand, the robustness value for testing the null hypothesis that the coefficient of EMR was zero (RV q = 1, alpha = 0.05) fell to 6.5%. This means that unobserved confounders that explained 6.5% of the residual variance from treatment and outcome variables were sufficiently strong to force the lower bound of the confidence interval (i.e., 5%) to zero. However, unobserved confounders that did not explain at least 6.5% of the residual variance from both the treatment and outcome variables were not strong enough to do so.

## 4. Discussion

According to the pre-pandemic evidence, living on small islands was usually considered an advantage when dealing with infectious diseases because of their small populations, geographical size, easier implementation of containment measures, and absence of land borders [5,6,7,8,9,10,18]. Nonetheless, during the management of the COVID-19 pandemic, studies from small island states in the Mediterranean Sea (i.e., Cyprus and Malta) [6,9,10], as well as reports from Iceland [6,9] and small island states in the Pacific Ocean [7], stressed a more diverse pattern. In smaller settings, not only can medical resources be rapidly depleted, but very small and episodic mistakes in the management of the pandemic (particularly with the lifting of NPIs) may be enhanced by high population density and the forced sharing of spaces and resources, quickly leading to substantial spreading of the pathogen [8,9].

However, our study suggests that Italian small islands were affected by the SARS-CoV-2 pandemic on a smaller scale than that reported at the national level, and particularly during its earlier stages [15]. EMR estimates for 2020 (−4.1%, 95% CI −12.3) and 2021 (4.1 vs. 4.6%, 95% CI −3.1 to 12.4) for the small islands were in fact substantially smaller than the corresponding national estimates of +18.8% for 2020 and +9.5% for 2021 [15]. In effect, during the “first wave” (i.e., March–April 2020), mortality rates were comparable to the expected ones, and such figures were quite consistent with available data from small island states of the Mediterranean Sea [9].

After a transition stage lasting from May to August 2020, Italy was involved in the “second wave” of the SARS-CoV-2 pandemic. During the “second wave”, the small islands exhibited a substantial increase in mortality rates, and again, such results were consistent with available international estimates, where this trend was explained by hasty relaxation of NPIs, including bans on national and international travel [6,9,10]. The removal of travel bans during summer 2020 and the subsequent resumption of tourism activities could likely explain such a trend. In fact, the small island states of the Mediterranean represent very popular tourist destinations, and their local economies also heavily depend on tourism. Not coincidentally, reports from Malta have associated the earlier stages of the “second wave” during the summer holidays with the local partial lifting of NPIs. More precisely, organized mass events that took place mid-July 2020 led to a significant spike in new cases and high community spread that anticipated by several weeks a similar trend in other European countries [6,9].

Nevertheless, the “second wave” on the Italian small islands still exhibited some interesting specificities. On the one hand, during the “second wave”, an increase in crude and excess mortality rates was identified compared to the “first wave”, mirroring at a lower level a similar trend at the national level. In turn, the increase in crude mortality rates and EMRs at the national level was well-correlated with notification rates for SARS-CoV-2 [19,20], with a trend that lasted until the inception of the vaccination campaign [15]. On the other hand, this stage of the second wave ended well before that reported both at the national and provincial levels [19,20,21]. More precisely, the small islands were spared from the peak in mortality rates that affected both the parent provinces and the nation as a whole in December 2020 [19,21,22].

Even though notification rates and mortality rates for COVID-19 remained substantially higher than those reported in the first wave, during the first half of 2021 and until the end of May 2021, actual mortality rates on the small islands were far lower than those expected. Only afterwards, and particularly during the warm season, did the Italian small islands exhibited a clear increase in mortality rates. Not coincidentally, while during 2020 the risk for all-cause deaths was similar to the figures for 2015–2019 (RR 0.992, 95% CI 0.933 to 1.056), the biphasic trend in mortality rates during 2021 for the small islands resulted in a clear surge (+9.6%) that was well below the estimates for the national level (RR 1.178, 95% CI 1.175 to 1.180).

This is particularly interesting for several reasons. First of all, during the “first wave”, Italy was severely affected by the SARS-CoV-2 pandemic [13,23], but while the following epidemic waves similarly involved all Italian regions, early hotspots of the pandemic were identified in the northern Italian regions of Lombardy, Piedmont, Veneto, Liguria, and Emilia Romagna, while regions from central and southern Italy were spared by the early implementation of NPIs and lockdown measures [13,24,25]. In fact, 34 out of 35 municipalities of the Italian small islands are associated with provinces from central Italy (La Spezia, Grosseto, and Latina), southern Italy (Foggia), or the main islands of Sicily (Messina, Trapani, Agrigento, and Palermo), and Sardinia (Sassari and South Sardinia). In other words, as suggested by estimates reported for parent provinces of Italian small islands, the latter may have benefited from a reduced background spreading of the pathogen rather than from the alleged advantages of small islands [26,27].

Even though the 2021 surge in EMRs on the small islands nearly halved that reported at the national level for the same time period [15], the overall rebound from the lower levels of calendar year 2020 was quite appreciable, and could be explained by a certain easing of NPIs and lockdown measures, as well as the resumption of inbound and outbound travel and tourism activities [6,10,18]. However, Italian small islands during 2021 still outperformed the national level in terms of EMRs, and this performance could be explained by the vigorous vaccination campaign that was implemented in order to achieve very high vaccination rates before the start of the warm (i.e., tourist) season of 2021 [28,29]. That intervention somehow mirrored the successful intervention rollout from Malta [18] and achieved the target of vaccinating all adults well before the beginning of the tourist season, i.e., by the end of May 2021 [30]. The holiday season was reasonably associated with the increased circulation of the pathogen, which in turn caused an increased number of infections and COVID-19 related deaths. However, the unprecedent number of new cases occurring since the winter season of 2021 and associated with variants of concern (VOC) Delta and Omicron (the latter initially isolated during November 2021, and prevalent since the end of December 2021) [31,32] only increased the mortality rates to a limited extent [27,33], reasonably because of the protective impact of the vaccines.

Another possible explanation for the better performance of Italian small islands during 2020 and 2021 compared to national estimates may be found in some further specificities of island communities. As recently suggested by Lu et al., communities characterized by higher acceptance of collective behavior are more likely to cope with NPIs, which in turn have been instrumental in slowing down pathogens such as SARS-CoV-2, particularly when effective vaccines were still unavailable [34,35]. From this point of view, smaller territories and enclosed communities, such as small islands, are characterized by a positive, shared attitude towards collectivism [36], or at least a better acceptance of NPIs through shared responsibility [34,36,37]. In fact, while acceptance of NPIs is quite difficult to objectivize because of the lack of population-level indicators, the quick and successful campaign to vaccinate the local population [18,29,30], not only on Italian small islands but also in other small island states [18], emphasizes a potential role for local and ancestral culture [36,37].

As expected, features of the SARS-CoV-2 pandemic were characterized as substantial effectors for EMR [12]. While notification rates and COVID-19 related deaths in all parent provinces positively predicted the EMRs on the small islands (i.e., the higher the notification rates and/or occurrence of deaths from COVID-19 infections, the higher the mortality rates among the inhabitants of small islands), features of hospital care were identified as negative effectors. In other words, the higher the number of patients that were admitted and treated in acute care facilities (i.e., hospitals and ICUs) at the provincial level, the lower the corresponding EMRs for small islands. A possible explanation may be found in the vulnerability of small islands to the rapid saturation of healthcare resources [6,7,38,39], an issue that is specifically associated with the SARS-CoV-2 pandemic [40,41]. Not only is Italy characterized by an uneven availability of healthcare resources, but its small islands are even more diverse and heterogenous from this perspective, and such specificities may contribute to explain the heterogenous results we were able to identify through our analyses [42,43]. Focusing on healthcare factors, and according to the report published by the Italian Health Ministry in 2016, well before the onset of the pandemic, only six hospitals were available to residents and tourists on the small islands (i.e., Portoferraio on the Isola d’Elba; Lacco Ameno on the Island of Ischia; Procida; Pantelleria; Lipari and La Maddalena), for a total of around 860 healthcare professionals and 255 hospital beds [11]. Moreover, only the Hospital of Ischia was equipped with an ICU. On all other islands, intensive care units were unavailable on the spot, requiring the transfer of patients to the mainland, which was otherwise required for the smaller and less populated islands. In other words, as the requirements of the pandemics have reasonably limited the potential on-time transfer of patients from the small islands to the provincial hubs, it is reasonable that the increase in mortality rates may have resulted not only from the direct effect of SARS-CoV-2 infections, but also from delays in proper medical management of other conditions [13,41,42]. During its various stages, the pandemic has forced healthcare providers to repeatedly restrain access to medical care and hospital services not only for patient safety (i.e., avoiding unnecessary exposures to environments that were potentially contaminated by SARS-CoV-2), but also because of the shortage of medical resources [40,41,44]. As a consequence, patients in actual need of medical treatment may not have received adequate and proper management, with an increased occurrence of indirect deaths.

*Limits*. Despite the potential interest, our study was affected by several limitations. First and foremost, it was an ecological study, and, therefore, it shared all of the implicit limits of this design, and particularly the potentially confounding factors represented by individual level variables [45,46,47].

The “ecological fallacy” in this specific case may have been amplified by the lack of detailed notification rates for SARS-CoV-2 positive cases at the municipal level. Particularly during the first stage of the pandemic, actual COVID-19 mortality may have been under-reported; according to a recent study on Italian data, missed diagnoses may have accounted for up to 96% of actual cases that occurred in the hotspot represented by the Italian province of Bergamo up to 25 September 2020 [48]. In this regard, because of the high infectivity rate and morbidity related to SARS-CoV-2 infection, despite the risk for their potential overestimation, mortality rates and particularly EMRs did represent a valuable and reliable proxy for the actual number of incident cases, particularly during the first year of the pandemic [9,10]. Contrarily, we cannot rule out the potential underestimation of deaths from more severe conditions (either for all-cause mortality or COVID-19 associated deaths) because of administrative constraints. In fact, in source data, cases are linked to the places of residence, not to the sites where the patients were treated.

Another bottleneck associated with the study design is represented by the quality of the data available from online sources (i.e., dashboards, platforms and other ministerial sites) [9]. For example, even though all-cause mortality appears at relatively low risk of reporting bias [49], mortality rates are calculated based on the number of residents, and this specific variable may be affected by substantial uncertainties, with individuals reporting a formal residence that does not reflect the actual one. Similarly, it should be stressed that detailed estimates of vaccination rates and actual adherence to NPIs in assessed areas were not available at the time of our study. Therefore, we cannot rule out that some better performances we were able to identify may have been associated with a more strict, local implementation of the NPI, and then by higher vaccination rates, which are quite difficult to characterize through an ecological design.

Moreover, as shown in Table A1 as well as Figure A1, Italian small islands are quite heterogenous in terms of basic demographics, including the share of active individuals, the distance from the main coasts, etc. In other words, several confounding factors not included in our analysis may have contributed to the results more strongly than the remoteness of the small islands. However, as suggested by sensitivity analysis, in an extreme scenario, even if confounders explained all remaining variation in the outcome, they would need to explain at least 6.5% of the residual variation to bring down the estimated effect to zero. Moreover, at least one confounder greater than 13.2% was necessary, otherwise such confounders could not explain the point estimates.

## 5. Conclusions

In conclusion, Italy’s small islands were extensively spared from the first wave of the SARS-CoV-2 pandemic, and were considerably less affected afterwards. Despite the heterogeneity among the retrieved estimates and the potential role of confounding factors represented by the local specificities of Italian small islands, which strongly recommend a cautious analysis of the reported data (e.g., actual distance from the mainland, share of active people among the general population, and conversely, the share of older age groups), several specificities of the small islands may have contributed to their better performance when compared with the national level and other small island communities, both in Europe and in the Pacific. However, the small islands cannot be simplistically defined as “safe havens” from COVID-19, not only because of the limited availability of medical resources, but also because the better performances we were able to identify may be associated with lockdown measures that are hardly compatible with local economies that are largely dependent on tourism. Specifically tailored preventive measures are, therefore, forcibly required in order to protect their resident populations. In this regard, available data suggest that achieving high vaccination rates may substantially reduce mortality rates in those populations.

## Figures and Tables

**Table 1:** Demographics of the 35 counties from Italian small Islands (2015–2021) compared to national estimates.

		2015 No. (%)	2016 No. (%)	2017 No. (%)	2018 No. (%)	2019 No. (%)	2020 No. (%)	2021 No. (%)	Diff. 2015–2021 %
Italy	Total Population	60,295,497 (100%)	60,163,712 (100%)	60,066,734 (100%)	59,937,769 (100%)	59,816,673 (100%)	59,641,488 (100%)	59,236,213 (100%)	−1.8%
	Total deaths	647,571 (100%)	615,261 (100%)	649,061 (100%)	633,133 (100%)	634,417 (100%)	740,317 (100%)	709,035 (100%)	+9.5%
	Male	29,228,315 (48.5%)	29,193,044 (48.5%)	29,178,654 (48.6%)	29,156,469 (48.6%)	29,131,195 (48.7%)	29,050,096 (48.7%)	28,866,226 (48.7%)	−1.2%
	Female	31,067,182 (51.5%)	30.970,668 (51.5%)	30,888,080 (51.4%)	30,781,300 (51.4%)	30,685,478 (51.3%)	30,591,392 (51.3%)	30,369,987 (51.3%)	−2.2%
	<20 years	11,172,613 (18.5%)	11,086,243 (18.4%)	10,986,232 (18.3%)	10,882,099 (18.2%)	10,745,563 (18.0%)	10,598,610 (17.8%)	10,493,558 (17.7%)	−6.1%
	20–39 years	14,033,525 (23.3%)	13,730,145 (22.8%)	13,483,326 (22.4%)	13,282,229 (22.2%)	13,113,139 (21.9%)	12,939,014 (21.7%)	12,712,317 (21.5%)	−9.4%
	40–59 years	18,295,227 (30.3%)	18,401,053 (30.6%)	18,454,245 (30.7%)	18,457,834 (30.8%)	18,445,702 (30.8%)	18,351,424 (30.8%)	18,142,711 (30.6%)	−0.8%
	60–79 years	12,837,919 (21.3%)	12,919,210 (21.5%)	13,036,923 (21.7%)	13,136,844 (21.9%)	13,215,981 (22.1%)	13,332,737 (22.4%)	13,408,810 (22.6%)	+4.4%
	≥ 80 years	3,956,213 (6.6%)	4,027,061 (6.7%)	4,106,008 (6.8%)	4,178,763 (7.0%)	4,296,288 (7.2%)	4,419,703 (7.4%)	4,478,817 (7.6%)	+13.2%
Small Islands	Total population	218,008 (0.4%)	217,391 (0.4%)	216,899 (0.4%)	216,569 (0.4%)	215,841 (0.4%)	215,200 (0.4%)	213,093 (0.4%)	−2.3%
	Total deaths	2206 (0.3%)	2115 (0.3%)	2224 (0.3%)	2250 (0.4%)	2118 (0.3%)	2247 (0.3%)	2409 (0.3%)	+9.2%
	Male	108,251 (49.7%)	108,125 (49.7%)	107,888 (49.7%)	107,686 (49.7%)	107,427 (49.7%)	107,080 (49.8%)	105,823 (49.7%)	−2.2%
	Female	109,757 (50.3%)	109,266 (50.3%)	109,011 (50.3%)	108,883 (50.3%)	108,414 (50.3%)	108,120 (50.3%)	107,270 (50.3%)	−2.3%
	<20 years	38,637 (17.7%)	38,066 (17.5%)	37,526 (17.3%)	36,998 (17.1%)	36,324 (16.8%)	35,067 (16.3%)	35,283 (16.6%)	−9.5%
	20–39 years	52,405 (24.0%)	51,227 (23.6%)	50,028 (23.1%)	49,183 (22.7%)	48,227 (22.3%)	47,375 (22.0%)	45,474 (21.3%)	−15.4%
	40–59 years	67,150 (30.8%)	67,530 (31.1%)	67,774 (31.2%)	67,797 (31.3%)	67,759 (31.4%)	67,477 (31.5%)	66,538 (31.2%)	−0.9%
	60–79 years	47,590 (21.8%)	47,979 (22.1%)	48,604 (22.4%)	49,241 (22.7%)	49,806 (23.1%)	50,618 (23.5%)	51,034 (23.9%)	+6.7%
	≥ 80 years	12,226 (5.6%)	12,589 (5.8%)	12,967 (6.0%)	13,350 (6.1%)	13,725 (6.4%)	14,148 (6.6%)	14,663 (6.9%)	+16.6%

**Table 2:** Yearly mortality rates on Italian small islands.

Province (Region)	County	Mortality Rates (Deaths per 100 Persons, [95% Confidence Intervals])		
		2015–2019	2020	2021
Foggia (Apulia)	Isole Tremiti	1.035 [0.657; 1.548]	0.891 [0.243; 2.265]	0.883 [0.241; 2.245]
Naples (Campania)	Anacapri	0.923 [0.823; 1.031]	0.906 [0.697; 1.158]	1.188 [0.946; 1.473]
	Barano d’Ischia	0.825 [0.747; 0.908]	0.708 [0.553; 0.892]	0.893 [0.717; 1.099]
	Capri	1.107 [0.999; 1.225]	1.164 [0.927; 1.443]	1.287 [1.035; 1.581]
	Casamicciola Terme	0.902 [0.811; 1.000]	0.805 [0.621; 1.027]	0.813 [0.625; 1.039]
	Forio	0.834 [0.773; 0.898	0.804 [0.677; 0.948]	0.926 [0.789; 1.079]
	Ischia	0.945 [0.888; 1.013]	0.967 [0.834; 1.115]	1.048 [0.910; 1.200]
	Lacco Ameno	0.927 [0.807; 1.059]	0.928 [0.675; 1.244]	1.140 [0.855; 1.489]
	Procida	1.174 [1.083; 1.272]	1.010 [0.826; 1.223]	1.289 [1.079; 1.528]
	Serrara Fontana	1.094 [0.932; 1.272]	0.941 [0.631; 1.348]	1.156 [0.807; 1.604]
Latina (Latium)	Ponza	1.245 [1.083; 1.435]	0.969 [0.664; 1.366]	1.119 [0.789; 1.539]
	Ventotene	1.097 [0.777; 1.502]	1.255 [0.578; 2.369]	1.637 [0.868; 2.905]
La Spezia (Liguria)	Porto Venere	1.767 [1.577; 1.973]	1.539 [1.151; 2.013]	1.621 [1.220; 2.110]
Sassari (Sardinia)	La Maddalena	1.033 [0.950; 1.122]	0.957 [0.783; 1.159]	1.237 [1.036; 1.465]
	Porto Torres	1.767 [1.577; 1.973]	1.166 [1.027; 1.318]	0.935 [0.810; 1.074]
South Sardinia (Sardinia)	Calasetta	1.214 [1.041; 1.408]	1.348 [0.956; 1.846]	1.581 [1.151; 2.117]
	Carloforte	1.460 [1.328; 1.602]	1.487 [1.196; 1.826]	1.376 [1.096; 1.705]
	Sant’Antioco	1.263 [1.172; 1.360]	1.402 [1.189; 1.641]	1.317 [1.110; 1.552]
Agrigento (Sicily)	Lampedusa e Linosa	0.781 [0.687; 0.884]	0.566 [0.397; 0.783]	0.836 [0.627; 1.093]
Messina (Sicily)	Lipari	0.950 [0.874; 1.031]	0.942 [0.780; 1.128]	1.069 [0.895; 1.266]
	Leni	1.054 [0.735; 1.463]	1.175 [0.509; 2.302]	1.022 [0.412; 2.094]
	Malfa	1.197 [0.906; 1.552]	1.005 [0.483; 1.841]	1.022 [0.491; 1.870]
	Santa Marina Salina	1.196 [0.892; 1.569]	1.634 [0.890; 2.726]	1.291 [0.646; 2.298]
Palermo (Sicily)	Ustica	1.138 [0.896; 1.424]	1.387 [0.824; 2.183]	0.938 [0.485; 1.632]
Trapani (Sicily)	Favignana	1.197 [1.055; 1.353]	1.397 [1.068; 1.759]	1.629 [1.274; 2.050]
	Pantelleria	1.197 [1.088; 1.314]	1.371 [1.119; 1.662]	1.412 [1.155; 1.708]
Grosseto (Tuscany)	Isola del Giglio	1.399 [1.133; 1.707]	1.620 [1.018; 2.443]	1.866 [1.211; 2.742]
Livorno (Tuscany)	Campo nell’Elba	1.133 [0.999; 1.280]	1.246 [0.948; 1.608]	1.151 [0.866; 1.499]
	Capoliveri	1.009 [0.869; 1.166]	0.897 [0.629; 1.240]	1.210 [0.890; 1.606]
	Capraia	1.226 [0.787; 1.818]	0.761 [0.157; 2.209]	1.042 [0.285; 2.646]
	Marciana	1.604 [1.371; 1.865]	1.288 [0.851; 1.869]	1.904 [1.358; 2.594]
	Marciana Marina	1.415 [1.186; 1.675]	1.047 [0.640; 1.612]	1.543 [1.035; 2.208]
	Portoferraio	1.186 [1.099; 1.278]	0.941 [0.775; 1.131]	1.168 [0.983; 1.378]
	Porto Azzurro	0.934 [0.792; 1.094]	1.014 [0.715; 1.395]	0.948 [0.661; 1.316]
	Rio	1.352 [1.178; 1.544]	1.457 [1.080; 1.922]	1.197 [0.857; 1.627]
	POOLED	1.105 [1.034; 1.181]	1.069 [0.983; 1.164]	1.180 [1.098; 1.269]

**Table 3:** Risk ratio for all-cause mortality on Italian small islands in 2020 and 2021 assuming estimates for 2018–2019 as a reference.

Province (Region)	County	Risk Ratio [95% Confidence Intervals]	
		2020	2021
Foggia (Apulia)	Isole Tremiti	0.861 [0.299; 2.478]	0.853 [0.297; 2.456]
Naples (Campania)	Anacapri	0.982 [0.750; 1.286]	1.288 [1.011; 1.640]
	Barano d’Ischia	0.859 [0.668; 1.104]	1.082 [0.861; 1.361]
	Capri	1.052 [0.829; 1.334]	1.162 [0.924; 1.462]
	Casamicciola Terme	0.893 [0.685; 1.164]	0.901 [0.690; 1.177]
	Forio	0.965 [0.805; 1.155]	1.111 [0.936; 1.318]
	Ischia	1.019 [0.871; 1.192]	1.104 [0.949; 1.284]
	Lacco Ameno	1.002 [0.725; 1.383]	1.231 [0.912; 1.660]
	Procida	0.860 [0.699; 1.058]	1.098 [0.910; 1.325]
	Serrara Fontana	0.860 [0.581; 1.274]	1.057 [0.736; 1.519]
Latina (Latium)	Ponza	0.776 [0.535; 1.126]	0.896 [0.632; 1.271]
	Ventotene	1.145 [0.560; 2.356]	1.526 [0.802; 2.906]
La Spezia (Liguria)	Porto Venere	0.871 [0.651; 1.165]	0.917 [0.689; 1.222]
Sassari (Sardinia)	La Maddalena	0.927 [0.752; 1.141]	1.197 [0.991; 1.445]
	Porto Torres	1.405 [1.223; 1.615]	1.128 [0.968; 1.314]
South Sardinia (Sardinia)	Calasetta	1.110 [0.783; 1.574]	1.302 [0.938; 1.809]
	Carloforte	1.018 [0.812; 1.276]	0.942 [0.746; 1.191]
	Sant’Antioco	1.110 [0.932; 1.321]	1.043 [0.871; 1.248]
Agrigento (Sicily)	Lampedusa e Linosa	0.725 [0.511; 1.027]	1.071 [0.797; 1.439]
Messina (Sicily)	Lipari	0.992 [0.814; 1.210]	1.125 [0.932; 1.359]
	Leni	1.114 [0.519; 2.391]	0.969 [0.432; 2.173]
	Malfa	0.840 [0.430; 1.640]	0.853 [0.437; 1.666]
	Santa Marina Salina	1.366 [0.760; 2.457]	1.080 [0.565; 2.063]
Palermo (Sicily)	Ustica	1.219 [0.731; 2.032]	0.824 [0.449; 1.511]
Trapani (Sicily)	Favignana	1.167 [0.883; 1.544]	1.361 [1.048; 1.767]
	Pantelleria	1.145 [0.925; 1.419]	1.180 [0.954; 1.459]
Grosseto (Tuscany)	Isola del Giglio	1.158 [0.731; 1.835]	1.334 [0.862; 2.065]
Livorno (Tuscany)	Campo nell’Elba	1.100 [0.829; 1.460]	1.016 [0.759; 1.360]
	Capoliveri	0.890 [0.623; 1.269]	1.199 [0.872; 1.649]
	Capraia	0.621 [0.188; 2.053]	0.850 [0.296; 2.435]
	Marciana	0.803 [0.536; 1.203]	1.187 [0.840; 1.677]
	Marciana Marina	0.740 [0.463; 1.180]	1.090 [0.732; 1.624]
	Portoferraio	0.793 [0.650; 0.968]	0.985 [0.822; 1.181]
	Porto Azzurro	1.085 [0.759; 1.552]	1.015 [0.704; 1.463]
	Rio	1.078 [0.792; 1.466]	0.885 [0.633; 1.238]
	TOTAL	0.992 [0.933; 1.056]	1.096 [1.049; 1.145]

**Table 4:** Risk Ratio for all-cause mortality on Italian small islands in 2020 and 2021 compared to parent provinces, assuming estimates for 2018–2019 as a reference.

Province	Risk Ratio [95% Confidence Intervals]	
	2020	2021
La Spezia	0.731 [0.569; 0.938]	0.828 [0.648; 1.057]
Livorno	0.875 [0.792; 0.966]	1.010 [0.921; 1.108]
Grosseto	1.087 [0.753; 1.570]	1.287 [0.918; 1.805]
La Latina	0.802 [0.607; 1.060]	0.929 [0.724; 1.193]
Napoli	0.898 [0.842; 0.956]	1.004 [0.947; 1.064]
Trapani	1.136 [0.991; 1.303]	1.139 [1.000; 1.298]
Palermo	1.102 [0.737; 1.649]	0.713 [0.424; 1.197]
Messina	1.037 [0.898; 1.198]	1.026 [0.894; 1.177]
Agrigento	0.703 [0.519; 0.951]	0.935 [0.736; 1.188]
Sassari	1.039 [0.946; 1.141]	1.014 [0.919; 1.118]
South Sardinia	0.946 [0.849; 1.053]	0.877 [0.785; 0.979]
Foggia	0.721 [0.296; 1.756]	0.731 [0.300; 1.782]
Total	0.939 [0.868; 1.756]	0.992 [0.943; 1.044]

**Table 5:** Standardized regression analysis predicting the excess mortality rates for Italian small islands (R^2^ = 0.109; adjusted R^2^ = 0.100). Estimates were corrected for main demographic factors (i.e., share of individuals aged 60 or more, sex, annual income) both on Italian small islands and in parent provinces (Note: SE = standard error; β = standardized estimate; B = unstandardized estimate).

	β	B	SE	95% Confidence Interval	*p*-Value
Constant		14.931	1.031	12.886; 16.940	<0.001
COVID-19 notification rates in parent province	0.469	0.882	0.168	0.551; 1.212	<0.001
COVID-19 related mortality in parent province	22.768	8.385	0.294	6.285; 39.250	0.007
COVID-19 related admissions in parent province	−11.208	3.278	−0.430	−17.651; −4.765	0.001
COVID-19 related admissions to ICUs in parent province	−59.700	19.710	−0.288	−98.444; −20.956	0.003

## Data Availability

Source data may be retrieved from the following databases: https://demo.istant.it (accessed on 20 March 2022) (Italian Institute of Statistics) and https://covid19.infn.it/iss/ (accessed on 20 March 2022) (National Institute for Nuclear Physics).

## Appendix A

**Table A1:** Characteristics of 35 municipalities from Italian small islands.

Municipality	Province	Region	Island(s)	Surface (km^2^)	Availability Of Hospital Infrastructure on the Main Island	Availability of ICU	Average Annual Income per Capita (€)	Total Population (2020) (No., % of All Small Islands)	Total Population (2021) (No., % of All Small Islands)	Total Deaths (2020) (No., % of All Small Islands)	Total Deaths (2021) (No., % of All Small Islands)
Anacapri	Naples	Campania	Capri	6.47	NO	NO	15,203.09	6954	6901	63	82
Barano d’Ischia	Naples	Campania	Ischia	10.96	YES	YES	19,493.79	10,027	9859	71	88
Calasetta	South Sardinia	Sardinia	Sant’Antioco	31.06	NO	NO	14,338.55	2819	2783	38	44
Campo nell’Elba	Livorno	Tuscany	Elba	55.79	YES	NO	24,036.92	4654	4691	58	54
Capoliveri	Livorno	Tuscany	Elba	39.56	YES	NO	16,807.08	4013	3884	36	47
Capraia	Livorno	Tuscany	Capraia	19.33	NO	NO	16,012.67	394	384	3	4
Capri	Naples	Campania	Capri	4.06	NO	NO	16,579.63	7042	6916	82	89
Carloforte	South Sardinia	Sardinia	San Pietro	51.10	NO	NO	16,422.76	5987	5960	89	82
Casamicciola Terme	Naples	Campania	Ischia	5.85	YES	NO	21,115.95	7949	7752	64	63
Favignana	Trapani	Sicily	Aegadian Islands (Favignana, Levanzo, Marettino, Formica, and Maraone)	37.45	NO	NO	14,190.88	4294	4359	60	71
Forio	Naples	Campania	Ischia	13.08	YES	NO	13,476.61	17,538	17,392	141	161
Ischia	Naples	Campania	Ischia	8.14	YES	NO	14,730.70	19,442	19,571	188	205
Isola del Giglio	Grosseto	Tuscany	Isola del Giglio	24.01	NO	NO	24,047.75	1358	1340	22	25
Isole Tremiti	Foggia	Apulia	Tremiti Islands (San Domino, San Nicola, Capraia, Cretaccio, and Pianosa)	3.18	NO	NO	18,341.20	449	453	4	4
La Maddalena	Sassari	Sardinia	La Maddalena Islands (La Maddalena, Caprera, Santo Stefano, Spargi, Budelli, Santa Maria, Razzoli)	52.01	YES	NO	16,465.69	10,865	10,674	104	132
Lacco Ameno	Naples	Campania	Ischia	2.08	YES	NO	15,726.12	4741	4648	44	53
Lampedusa e Linosa	Agrigento	Sicily	Pelagie Islands (Lampedusa, Linosa, Lampione)	25.22	NO	NO	17,774.39	6360	6337	36	53
Leni	Messina	Sicily	Salina	8.79		NO	15,227.14	681	685	8	7
Lipari	Messina	Sicily	Aeolian Islands (Lipari, Vulcano, Panarea, Stromboli, Filicudi, and Alicudi)	89.72	YES	NO	13,926.59	12,415	12,351	117	132
Malfa	Messina	Sicily	Salina	8.74	NO	NO	14,975.85	995	979	10	10
Marciana	Livorno	Tuscany	Elba	45.45	YES	NO	15.118.69	2096	2048	27	39
Marciana Marina	Livorno	Tuscany	Elba	5.86	YES	NO	15,685.86	1911	1880	20	29
Pantelleria	Trapani	Sicily	Pantelleria	84.53	YES	NO	16,770.52	7441	7367	102	104
Ponza	Latina	Latium	Pontian Islands (Ponza, Palmarola, Gavi, Zannone)	10.16	NO	NO	15,590.95	3301	3306	32	37
Porto Azzurro	Livorno	Tuscany	Elba	13.33	YES	NO	15,564.11	3650	3692	37	35
Porto Torres	Sassari	Sardinia	Asinara, Isola Piana	104.41	NO	NO	14,101.53	21,618	21,274	252	199
Porto Venere	La Spezia	Liguria	Palmaria	7.66	NO	NO	17,748.37	3380	3331	52	54
Portoferraio	Livorno	Tuscany	Elba	48.48	YES	NO	16,899.45	11,903	11,897	112	139
Procida	Naples	Campania	Procida, Vivara	4.26	YES	NO	18,264.82	10,295	10,162	104	131
Rio	Livorno	Tuscany	Elba	36.52	YES	NO	19,467.12	3363	3341	49	40
Sant’Antioco	South Sardinia	Sardinia	Sant’Antioco	87.90	NO	NO	16,620.89	10,842	10,705	152	141
Santa Marina Salina	Messina	Sicily	Salina	8.78	NO	NO	18,668.81	857	852	14	11
Serrara Fontana	Naples	Campania	Ischia	6.44	YES	NO	19,135.50	3083	3027	29	35
Ustica	Palermo	Sicily	Ustica	8.65	NO	NO	17,225.64	1298	1280	18	12
Ventotene	Latina	Latium	Ventotene, Santo Stefano	1.75	NO	NO	17,548.52	717	717	9	12
